# Community Mitigation of COVID-19 and Portrayal of Testing on TikTok: Descriptive Study

**DOI:** 10.2196/29528

**Published:** 2021-06-10

**Authors:** Corey H Basch, Jan Mohlman, Joseph Fera, Hao Tang, Alessia Pellicane, Charles E Basch

**Affiliations:** 1 Department of Public Health William Paterson University Wayne, NJ United States; 2 Department of Psychology William Paterson University Wayne, NJ United States; 3 Department of Mathematics Lehman College The City University of New York Bronx, NY United States; 4 Department of Health and Behavior Studies Teachers College Columbia University New York, NY United States

**Keywords:** TikTok, social media, COVID-19, testing, disgust, anxiety, content analysis, communication, infodemiology, infoveillance, public health, digital public health, digital health, community mitigation

## Abstract

**Background:**

COVID-19 testing remains an essential element of a comprehensive strategy for community mitigation. Social media is a popular source of information about health, including COVID-19 and testing information. One of the most popular communication channels used by adolescents and young adults who search for health information is TikTok—an emerging social media platform.

**Objective:**

The purpose of this study was to describe TikTok videos related to COVID-19 testing.

**Methods:**

The hashtag #covidtesting was searched, and the first 100 videos were included in the study sample. At the time the sample was drawn, these 100 videos garnered more than 50% of the views for all videos cataloged under the hashtag #covidtesting. The content characteristics that were coded included mentions, displays, or suggestions of anxiety, COVID-19 symptoms, quarantine, types of tests, results of test, and disgust/unpleasantness. Additional data that were coded included the number and percentage of views, likes, and comments and the use of music, dance, and humor.

**Results:**

The 100 videos garnered more than 103 million views; 111,000 comments; and over 12.8 million likes. Even though only 44 videos mentioned or suggested disgust/unpleasantness and 44 mentioned or suggested anxiety, those that portrayed tests as disgusting/unpleasant garnered over 70% of the total cumulative number of views (73,479,400/103,071,900, 71.29%) and likes (9,354,691/12,872,505, 72.67%), and those that mentioned or suggested anxiety attracted about 60% of the total cumulative number of views (61,423,500/103,071,900, 59.59%) and more than 8 million likes (8,339,598/12,872,505, 64.79%). Independent one-tailed *t* tests (α=.05) revealed that videos that mentioned or suggested that COVID-19 testing was disgusting/unpleasant were associated with receiving a higher number of views and likes.

**Conclusions:**

Our finding of an association between TikTok videos that mentioned or suggested that COVID-19 tests were disgusting/unpleasant and these videos’ propensity to garner views and likes is of concern. There is a need for public health agencies to recognize and address connotations of COVID-19 testing on social media.

## Introduction

Testing is necessary for estimating community positivity rates and tracking the ongoing distribution of active COVID-19 cases by person, place, and time factors. The ongoing tracking of the distribution of the disease is one of the key strategies for community mitigation [1]. Testing is therefore centrally important in COVID-19 prevention. First identified in December 2019, COVID-19 quickly reached pandemic status, and the disease has had devastating consequences across the globe [2]. As of May 8, 2021, there have been 157,087,436 confirmed cases of COVID-19 and 3,274,092 deaths reported worldwide [2].

Due to the highly contagious nature of COVID-19, the importance of rapid and accurate testing cannot be understated [3]. The two main categories of COVID-19 tests are diagnostic tests for detecting current infection and antibody tests for detecting prior infection [4,5]. Molecular tests, including real-time reverse-transcription polymerase chain reaction tests and nucleic acid amplification tests, detect genetic material in SARS-CoV-2, while antigen tests detect proteins on the virus’s surface; both tests are used to diagnose active infections [5,6]. In contrast to molecular and antigen tests, antibody and serology testing uses a blood sample to detect antibodies that were produced from a previous SARS-CoV-2 infection and cannot be used to diagnose current infection. Further, it is still unclear if the presence of antibodies results in future immunity to COVID-19 [4]. At present, antigen tests provide the fastest results (less than 1 hour), but a negative test cannot definitively rule out an active infection. Results from molecular tests are more accurate, and they may become available within 1 day or take up to 1 week; antibody test results are generally available within 1 to 3 days [7,8]. Improvements in diagnostic testing are evolving to identify active cases more rapidly and may help inform treatment [5-8]. At present, emergency use authorizations have been provided by the US Food and Drug Administration for different types of COVID-19 tests [9-11].

In molecular and antigen testing, samples are attained mostly via nasal or nasopharyngeal swabs, and a few molecular tests use saliva [5,12]. Molecular testing is the most accurate method for diagnosing current SARS-CoV-2 infections, as it can be used to detect the unique genetic sequence of SARS-CoV-2 [13]. Various companies and labs have developed a variety of molecular diagnostic tests, which may result in variation in the process of testing [14]. If an antigen test indicates a negative result (suggesting that there is no current infection), a molecular test can be used to avoid false-negative results [5,15]. At present, there is no COVID-19 test that is always 100% accurate [16], and individuals should choose a test based on their own situations and health care providers’ recommendations [17].

Conducting rapid COVID-19 tests in high volume can lead to the timely identification and treatment of people with SARS-CoV-2 infection and help prevent disease transmission. It has been suggested that people with mild or no symptoms can transmit the virus [18], which further highlights the importance of the early identification and containment of people who are capable of transmitting disease. Testing can also help identify individuals who have been in contact with people with COVID-19, so that they can all be treated quickly. COVID-19 tests are an essential element of addressing the pandemic by helping researchers track the distribution of the disease, understand the contagious characteristics of the disease, and trace contacts [19,20]. Consequently, organizations such as the Centers for Disease Control and Prevention (CDC), Food and Drug Administration, and World Health Organization have been attempting to increase the availability and accessibility of COVID-19 tests.

The negative perceptions surrounding COVID-19 testing have persisted. These negative attitudes may stem from the discomfort of the testing process [21], compulsory testing, a fear of isolation, a worry of movement restriction [20,22,23], misconceptions about the virus or bad medical memory [24], fear or anxiety [25], and concerns related to accuracy [26,27]. Public fear and uncertainty have been recurrent themes of the COVID-19 pandemic.

Coronaphobia is defined as “as an excessive triggered response of fear of contracting the virus causing COVID-19, leading to accompanied excessive concern over physiological symptoms, significant stress about personal and occupational loss, increased reassurance and safety seeking behaviors, and avoidance of public places and situations, causing marked impairment in daily life functioning” [28]. It may contribute to fear, disgust, concern, and other negative attitudes toward COVID-19 tests. Although there is an abundance of information on COVID-19 being distributed, social media has been particularly popular in this context [29-32]. Social media has been found to have a significant potential influence on sources of information that are used by consumers, which in turn may influence perceptions and behaviors regarding COVID-19 prevention [33]. Despite this, research related to how COVID-19 testing is portrayed and discussed on social media is scarce.

One of the most popular social media platforms is TikTok, which has 689 million users across 150 countries and 100 million users in the United States [34]. In the United States, TikTok is most commonly used by those aged 10 to 19 years (32.5%), followed by 20- to 29-year-olds (29.5%) [34]. TikTok was the most downloaded mobile app in 2020 [35]. Similar to other social media platforms, there is an abundance of information related to COVID-19 on TikTok, including information that promotes health [36] and that which is misinformative [37]. It was found that 28% of TikTok users share COVID-19–related news [34]. Given the COVID-19 “infodemic,” the popularity of TikTok, and the lack of related research, the purpose of this study was to describe content related to COVID-19 testing on TikTok.

## Methods

This was a cross-sectional descriptive study that paralleled methods that were used in prior studies of TikTok [36-38]. By using discover mode and the hashtag #covidtesting, a sample of 100 videos was drawn in February 2021. This hashtag had the greatest number of views related to COVID-19 testing (around 191.2 million views at the time of this study). The content characteristics coded for each video included mentioning or displaying viral tests, antibody tests, nasal swabs, and test results; mentioning or suggesting that tests were disgusting/unpleasant and mentioning or suggesting anxiety, quarantine, and COVID-19 symptoms; and using music, dance or humor. In addition, the number and percentage of views, likes, and comments were recorded, and whether a video quoted a scientifically credible source was noted. All 100 videos were coded by a single reviewer (AP), while a second reviewer (CHB) coded a 10% random sample to demonstrate interrater reliability. The two coders’ opinions differed on only 2 of the 150 data points (κ=0.97). The analysis was completed by using Microsoft Excel and included descriptive statistics and independent one-tailed *t* tests and chi-square tests (α=.05). As per the Institutional Review Board (IRB) at William Paterson University, studies without human subjects are not reviewed. The IRB at Teachers College, Columbia University deemed this study exempt from review.

## Results

The 100 videos garnered more than 103 million views; 111,000 comments; and 12.8 million likes (Table 1). Only two content characteristics appeared in a majority of the videos—mentioning or displaying a viral test (88/100, 88%) or nasal swab (61/100, 61%). The 88 videos that mentioned or displayed a COVID-19 test garnered over 95% of the total cumulative number of views (98,303,500/103,071,900, 95.37%), over 92% of the total number of comments (102,898/111,817, 92.02%), and over 94% of the total number of likes (12,127,252/12,872,505, 94.21%), while the 61 videos that mentioned or displayed a nasal swab accumulated over 62% of the total number of views (64,010,600/103,071,900, 62.10%), over 76% of the total number of comments (85,222/111,817, 76.22%), and over 59% of the total number of likes (7,655,082/12,872,505, 59.47%). Few videos used dance (n=3) and mentioned or displayed an antibody or blood test (n=2), and only 1 video explicitly cited a scientifically credible source (Johns Hopkins Coronavirus Resource Center).

**Table 1:** Views, comments, and likes for COVID-19 testing–related videos on TikTok based on observed content characteristics.

Characteristic	Videos (N=100), n	Views (N=103,071,900), n (%)	Comments (N=111,817), n (%)	Likes (N=12,872,505), n (%)
Mentioned or displayed a viral test	88	98,303,500 (95.37)	102,898 (92.02)	12,127,252 (94.21)
Mentioned or displayed a nasal swab	61	64,010,600 (62.10)	85,222 (76.22)	7,655,082 (59.47)
Mentioned or displayed test results	30	22,207,200 (21.55)	17,960 (16.06)	1,550,191 (12.04)
Mentioned or suggested that tests are disgusting/Unpleasant	44	73,479,400 (71.29)	78,625 (70.32)	9,354,691 (72.67)
Mentioned or suggested anxiety	44	61,423,500 (59.59)	71,088 (63.58)	8,339,598 (64.79)
Mentioned or suggested quarantine	13	3,990,500 (3.87)	4835 (4.32)	783,443 (6.09)
Mentioned or suggested COVID-19 symptoms	11	26,825,700 (26.03)	27,050 (24.19)	2,523,617 (19.60)
Used music	47	29,610,600 (28.73)	43,148 (38.59)	4,186,839 (32.53)
Used humor	33	38,338,200 (37.20)	49,392 (44.17)	6,447,726 (50.09)

Even though only 44 videos mentioned or suggested that tests are disgusting/unpleasant and 44 mentioned or suggested anxiety, those that portrayed tests as disgusting/unpleasant garnered over 70% of the total cumulative number of views (73,479,400/103,071,900, 71.29%) and likes (9,354,691/12,872,505, 72.67%), and those that mentioned or suggested anxiety attracted around 60% of the total cumulative number of views (61,423,500/103,071,900, 59.59%) and more than 8 million likes (8,339,598/12,872,505, 64.79%). Independent one-tailed *t* tests (significance was set at *P*<.05) showed that suggesting anxiety had no statistical association with a video receiving views (*P*=.13), comments (*P*=.12), or likes (*P*=.10). However, mentioning or suggesting that testing was disgusting/unpleasant was associated with garnering views and likes, indicating that videos portraying a COVID-19 test as disgusting/unpleasant were more likely to receive a higher number of views and likes. Of the 33 videos that used humor, 23 (69.7%) mentioned or suggested that tests were disgusting/unpleasant (*P*<.001) and 23 (69.7%) mentioned or suggested anxiety (*P*<.001); hence, using humor was statistically associated with whether a video portrayed tests as disgusting/unpleasant and mentioned or suggested anxiety.

## Discussion

There is a need for public health agencies to recognize and address the connotations associated with COVID-19 testing on social media. We found that mentioning or suggesting that COVID-19 tests are disgusting/unpleasant was associated with the number of TikTok views and likes. Although the cross-sectional nature of this study precludes causal inferences, our data suggest that portraying a test as a disgusting/unpleasant medical procedure may generate viewer interest and promote the endorsement of videos. This finding was unexpected and troubling but perhaps not surprising.

The behavioral immune system theory consists of psychological constructs and processes that are hypothesized to encourage disease prevention behaviors [39-41]. One component—disgust sensitivity—has been consistently associated with certain disease prevention behaviors [42], including germ aversion and pathogen disgust related to COVID-19 prevention [39,41]. Yet in our study, we found that mentioning or suggesting disgust/unpleasantness in TikTok videos about COVID-19 testing was associated with the cumulative number of views and likes.

The methodology for COVID-19 tests vary according to the test type, which in turn may influence perceptions regarding disgust/unpleasantness. For example, nasal swab testing involves inserting a soft nasal swab into the nostril (length of insertion: 1.5 cm for an anterior nasal swab and 2 cm for a midturbinate specimen). The CDC guidelines for nasal swab testing are as follows: “Slowly rotate the swab, gently pressing against the inside of your nostril at least 4 times for a total of 15 seconds. Get as much nasal discharge as possible on the soft end of the swab” [4]. This should be repeated for each nostril. It is important to note that a few molecular tests involve gathering saliva in a tube [5].

Disgust is known as an emotional precursor of avoiding unpleasant medical procedures [43]. It can be argued that disgust could potentially unite like-minded people in efforts to promote health [40]. From an evolutionary standpoint, disgust can be conceptualized as information that is best shared with others, given that the well-being of society hinges upon the mass avoidance of harmful experiences and aversive stimuli [44]. In videos that mentioned or suggested that COVID-19 tests are a disgusting/unpleasant experience, individuals might either intentionally or inadvertently endorse avoidance. It is thus plausible that viewers of TikTok videos may form an ad hoc community of sorts that is in agreement with avoiding COVID-19 testing. It may be this sense of community, and not the videos themselves, that are associated with the number of views and likes [45].

Another factor that may generate a high number of likes is the disclosure of an aversive experience, which may provide a sense of relief. Perhaps this is even more the case when this point of view is shared among members of a group, such as the many viewers of a TikTok video. In summary, a shared dislike of COVID-19 testing potentially gives rise to the positive emotions that are associated with membership in a group [46], leading to the liking of videos. It seems probable that a different, unidentified subset of TikTok viewers did not like the videos on COVID-19 testing. It has long been recognized that phobias are prone to informational transmission, meaning that emotions such as fear and disgust are propagated and exacerbated by way of news stories, media coverage, and similar informational sources [47]. Perhaps those who did not like COVID-19 testing–related videos on TikTok avoided these videos due to their preexisting fears of the medical procedure, knowing that viewing these videos could lead to the further exacerbation of coronaphobia [28,48].

The limitations of this study include its cross-sectional design, the small sample size, the restricted scope of data coded, and the inability to account for the algorithms that yielded the sample. A cross-sectional design is particularly important when it comes to social media studies, such as studies on TikTok, due to the constantly changing nature of information on this widely used communication channel. In addition, we do not know the extent to which the results would be different had a larger sample of videos been included. However, at the time of this study, the collective number of views for videos with the hashtag #covidtesting was 191.2 million, and the videos included in this study had over 103 million views. This study only included a limited range of coded information, which restricted the inferences that could be drawn. For example, there was no distinction between self-administered testing and clinical testing, as this would have required making assumptions, which could lead to inaccuracies. The algorithms that produced the sample are unavailable, as are the details on how these algorithms may change over time. Further, while view counts are registered on TikTok, there is no method for determining if a viewer observed the entirety of the video. Despite these limitations, this is the first study that examines TikTok videos related to COVID-19 testing and illustrates the importance of continuing research on this topic.

## Abbreviations

CDC: Centers for Disease Control and Prevention
IRB: Institutional Review Board
