# Correction: Community Mitigation of COVID-19 and Portrayal of Testing on TikTok: Descriptive Study

**DOI:** 10.2196/31542

**Published:** 2021-06-29

**Authors:** Corey H Basch, Jan Mohlman, Joseph Fera, Hao Tang, Alessia Pellicane, Charles E Basch

**Affiliations:** 1 Department of Public Health William Paterson University Wayne, NJ United States; 2 Department of Psychology William Paterson University Wayne, NJ United States; 3 Department of Mathematics Lehman College The City University of New York Bronx, NY United States; 4 Department of Health and Behavior Studies Teachers College Columbia University New York, NY United States

In “Community Mitigation of COVID-19 and Portrayal of Testing on TikTok: Descriptive Study” (JMIR Public Health Surveill 2021;7(6):e29528), one error was noted.

Due to a system error, the name of one author, Hao Tang, was replaced with the name of another author on the paper, Alessia Pellicane. In the originally published paper, the order of authors was listed as follows:

Corey H Basch, Jan Mohlman, Joseph Fera, Alessia Pellicane, Alessia Pellicane, Charles E Basch

This has been corrected to:

Corey H Basch, Jan Mohlman, Joseph Fera, Hao Tang, Alessia Pellicane, Charles E Basch

In the originally published paper, the ORCID of author Hao Tang was incorrectly published as follows:

0000-0002-8220-6584

This has been corrected to:

0000-0003-3002-1374

The correction will appear in the online version of the paper on the JMIR Publications website on June 29, 2021, together with the publication of this correction notice. Because this was made after submission to PubMed, PubMed Central, and other full-text repositories, the corrected article has also been resubmitted to those repositories.

